# Will Sirtuin 2 Be a Promising Target for Neuroinflammatory Disorders?

**DOI:** 10.3389/fncel.2022.915587

**Published:** 2022-06-22

**Authors:** Zhang Fan, Li Bin

**Affiliations:** ^1^Beijing Key Laboratory of Basic Research With Traditional Chinese Medicine (TCM) on Infectious Diseases, Beijing Institute of Chinese Medicine, Beijing Hospital of TCM, Capital Medical University, Beijing, China; ^2^Beijing Key Laboratory of Acupuncture Neuromodulation, Acupuncture and Moxibustion Department, Beijing Hospital of TCM, Capital Medical University, Beijing, China

**Keywords:** sirtuin 2 (SIRT2), deacetylation, neuroinflammatory disorders, inflammatory, neuroprotection, promising target

## Abstract

Neuroinflammatory disorder is a general term that is associated with the progressive loss of neuronal structure or function. At present, the widely studied diseases with neuroinflammatory components are mainly divided into neurodegenerative and neuropsychiatric diseases, namely, Alzheimer’s disease, Parkinson’s disease, depression, stroke, and so on. An appropriate neuroinflammatory response can promote brain homeostasis, while excessive neuroinflammation can inhibit neuronal regeneration and damage the central nervous system. Apart from the symptomatic treatment with cholinesterase inhibitors, antidepressants/anxiolytics, and neuroprotective drugs, the treatment of neuroinflammation is a promising therapeutic method. Sirtuins are a host of class III histone deacetylases, that require nicotinamide adenine dinucleotide for their lysine residue deacetylase activity. The role of sirtuin 2 (SIRT2), one of the sirtuins, in modulating senescence, myelin formation, autophagy, and inflammation has been widely studied. SIRT2 is associated with many neuroinflammatory disorders considering it has deacetylation properties, that regulate the entire immune homeostasis. The aim of this review was to summarize the latest progress in regulating the effects of SIRT2 on immune homeostasis in neuroinflammatory disorders. The overall structure and catalytic properties of SIRT2, the selective inhibitors of SIRT2, the relationship between immune homeostasis and SIRT2, and the multitasking role of SIRT2 in several diseases with neuroinflammatory components were discussed.

## Introduction

With aging populations worldwide, the central nervous system (CNS) diseases associated with aging have been on the rise (Hou et al., 2019). Neurological diseases such as depression and stroke have also been increasing in young and middle-aged people (Pascoe and Parker, 2019; Putaala, 2020). Recent studies have found that the occurrence and development of these diseases have a common feature related to neuroinflammation, which generally refers to the noxious effects caused by immunological activation of microglia and astrocytes in various diseases of the CNS (Kwon and Koh, 2020; Griciuc and Tanzi, 2021). It is a physiological response of the immune system to harmful stimuli (Akbari et al., 2020; Jia et al., 2021). In response to harmful stimuli, neutrophils, monocytes/macrophages, different subtypes of T cells, and other inflammatory cells are commonly activated to maintain the immune system’s balance (Maida et al., 2020). An appropriate neuroinflammatory response can promote brain homeostasis, while excessive neuroinflammation can inhibit neuronal regeneration (Yang B. M. et al., 2019). An immune imbalance has been considered to be the mechanism of the deterioration of a variety of CNS diseases, including Alzheimer’s disease (AD) (Armstrong, 2019), Parkinson’s disease (PD) (Schonhoff et al., 2020), depression (Hayley et al., 2021), stroke (Iadecola et al., 2020), and so on. Thus, restoring the balance of the immune homeostasis has been suggested as a new treatment approach for diseases with neuroinflammatory components. These biologic molecules of immune regulation have moved toward the focus of research. Based on the specific effects on inflammatory factors, sirtuin 2 (SIRT2) has attracted much academic attention. We further reviewed the specific and detailed role of SIRT2 in neuroinflammatory disorders in the CNS.

**FIGURE 1:**
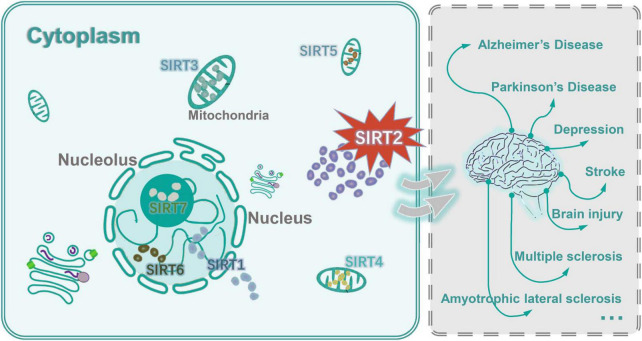
Subcellular location of 7 mammalian SIRTs (SIRT1–7) and SIRT2 related to neuroinflammatory disorders. SIRT1, SIRT6, and SIRT7 were found in the nucleus of the cell. Under certain conditions, SIRT1 can be transported from the nucleus to the cytoplasm. SIRT6 was also found in heterochromatin and the endoplasmic reticulum. SIRT7 was located at the nucleolus. SIRT3, SIRT4, and SIRT5 were found in the mitochondria and contribute to oxidative stress alleviation by regulating the activity of specific metabolic enzymes. SIRT3 is moved between the nucleus and mitochondria under cellular stress. The main site of SIRT2 was the cytoplasm, but in some phases of the cell cycle it was also found in the nucleus. SIRT2 was involved in the pathogenesis and progression of neuroinflammatory disorders.

Sirtuin 2 is predominantly found in the cytoplasm of cells in the (CNS), where it balances the whole immune homeostasis *via* the nicotinamide adenine dinucleotide (NAD)-dependent histone deacetylases (HDACs) (Gu et al., 2020; Hong et al., 2020; Zhang et al., 2021a; Figure 1). SIRT2 has a generic catalytic core domain like other Sirtuin family members and unique N-terminal and C-terminal sequences (Yang et al., 2020). Substrate and the catalytic core domain are vital during SIRT2 deacetylation (Minten et al., 2021). Therefore, SIRT2 participates in many biological processes by catalyzing the deacetylation of various substrates, such as senescence, myelin formation, autophagy, and inflammation (Puigoriol-Illamola et al., 2020; Lu et al., 2021). Yet, the exact role of SIRT2 in the immune regulation of neuroinflammatory disorders is still not fully understood. This review summarized the latest advances in SIRT2 in neuroinflammatory disorders, and the role of SIRT2 in balancing the immune homeostasis and inflammation in the CNS.

## Structure and Function of Sirtuin 2

Sirtuin 2, an NAD+ dependent protein deacetylase, is the only sirtuin protein that is predominantly found in the cytoplasm. Yet, SIRT2 is also found in the mitochondria and nucleus (Lin et al., 2021).

### Overall Structure of Sirtuin 2

**FIGURE 2:**
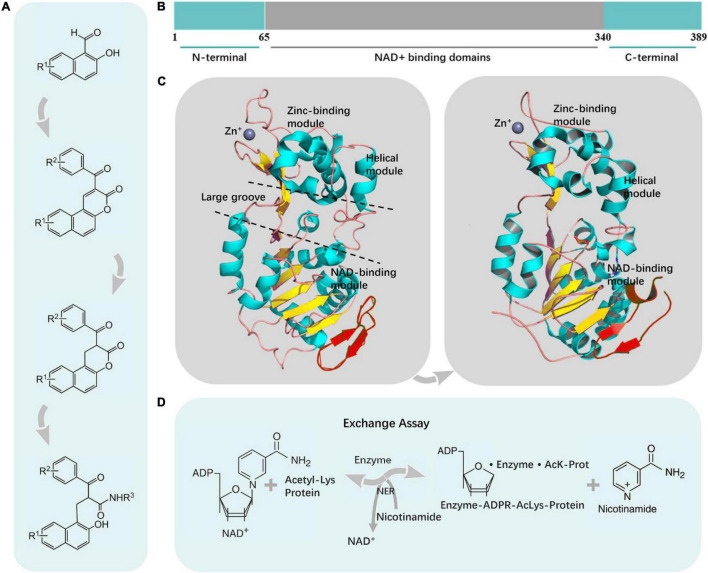
The overall structure of SIRT2. **(A)** Synthesis of SIRT2 inhibitors. **(B)** Schematic representation of human SIRT2. The conserved large catalytic domain was shown in gray. Numbers referred to amino acid residues in the proteins. **(C)** SIRT2 consisted of two domains that were connected by several conserved loops. **(D)** The SIRT2 enzymatic reaction was composed of three sequential steps, two of which were reversible and the final one irreversible.

The human SIRT2 gene, mapped at chromosome 19q13.2, is composed of 16 exons and spans a region of 20,960 bp (Yang W. T. et al., 2017; Yang Y. et al., 2017). SIRT2 has a 323 amino acid catalytic core, which is flanked by a 19-residue N-terminal and a C-terminal helical extension (Li et al., 2015; Figure 2B). Two isoforms of the SIRT2 protein are encoded: a 389-aa protein with a predictive molecular weight of 43.2 kDa and an isoelectric point of 5.22 is encoded by isoform 1, a 352-aa protein with a predictive molecular weight of 39.5 kDa, and an isoelectric point of 6.05 is encoded by isoform 2, which lacks the first three exons (Alcaín and Villalba, 2009).

The catalytic core has an elongated shape containing two domains, namely, a larger domain (residues 53–89, 146–186, and 241–356) and a smaller domain (residues 90–145 and 187–240) (Finnin et al., 2001). The former is a variant of the Rossmann fold, which is present in many diverse NAD(H)/NADP(H) binding enzymes (Hou et al., 2014). And the latter is composed of a helical module and a zinc-binding module (Carafa et al., 2012; Karaman et al., 2018). The two domains are connected by a hinge region that consists of four crossovers of the polypeptide chain (Finnin et al., 2001). At the interface of the two domains, the four crossovers and three loops of the large domain form a large groove (Liu Y. M. et al., 2019; Figure 2C).

The larger domain consists of six β strands and eight α helices (North and Verdin, 2004). The β strands form a parallel β sheet, and the α helices pack against the β sheet (Finnin et al., 2001). The Rossmann fold contains various features of a typical NAD+ binding site, such as a conserved Gly-X-Gly sequence important for NAD+ phosphate binding, a charged amino acid residue responsible for ribose group binding, and a small pocket to accommodate an NAD+ molecule (Satoh et al., 2011; Zhang X. et al., 2021; Figure 2C).

The smaller domain contains two structural modules that stem from two insertions in the Rossmann fold of the large domain (North and Verdin, 2004). The first insertion comprises four α helices that fold to form the helical module (Finnin et al., 2001). The second insertion consists of a β sheet, an α helix, and a zinc atom coordinated by four Cys residues (Finnin et al., 2001). A pocket in the helical module lines with hydrophobic residues and could intersect with the large groove between the larger and smaller domains, which is a class-specific binding site for proteins. The zinc-binding module has a topology, that mediates the interactions between protein and protein in diverse proteins (Figure 2C).

The SIRT2 enzymatic reaction consists of three sequential steps. Two of them are reversible, and one, the final step, is irreversible. The enzyme catalyzes the deacetylation of an acetylated substrate with the NAD+. The Acetyl Lys protein is an acetylated substrate. These three steps are involved in the binding of NAD+ and an acetylated lysine substrate; the formation of an enzyme-ADP-Ribose (ADPR)-acetylated lysine (AcLys-protein) intermediate (enzyme-ADPR-AcLys-protein) with the release of nicotinamide (Nic); and at last, 2′-O-acetyl-ADP-ribose (AADPR) and the deacetylated protein release. The reversibility of the second step is the basis of the NER assay, as only in this way the excess of labeled nicotinamide can reverse the reaction, and the labeled NAD+ can be produced *de novo* (Figure 2D).

### Catalytic Properties of Sirtuin 2

The sirtuin family is an NAD+ dependent HDACs family, which has been highly conserved during its evolution (Dai et al., 2018). SIRT2 is a member of the Sirtuin family that mediates transcriptional silencing at telomeres, mating-type loci, and ribosomal gene clusters (Finnin et al., 2001). Its major characteristic is that it can realize deacetylation of acetyllysine substrate (Wang et al., 2019). NAD+ is the basis of this catalytic reaction. Moreover, it influences the direct response of SIRT2 to the changes in intracellular metabolism (Jêśko et al., 2017). As a redox-active metabolite, NAD+ can be converted from NADH or into NADH. Thus, the ratio of NAD+/NADH affects the activity of SIRT2 (Ren et al., 2020). In addition, the activity of SIRT2 is also influenced by nicotinamide, which is the product of the deacetylation process (Ma et al., 2014; Li et al., 2018).

During the deacetylation reaction mediated by SIRT2, NAD+, SIRT2, and acetyllysine substrate interact to produce three products, i.e., the nicotinamide, the deacetylated substrate, and AADPR (Sedlackova and Korolchuk, 2020). The detailed steps of the NAD+ dependent deacetylation are roughly divided into three parts (Tatone et al., 2015; Grabowska et al., 2017; Carafa et al., 2019; Lewis et al., 2019): (1) the first part is mediated by NAD+, where SIRT2 recognizes and binds to the substrate; during this process, the NAD+, SIRT2, and substrate can form a ternary complex. Also, the combination between NAD+ and SIRT2 is related to the NAD+ binding site on the Rossmann fold of SIRT2; (2) the second part mainly includes NAD+ releasing the free nicotinamide and the activated ADP-ribose, which is realized by SIRT2 cleaving the glycosidic bond between nicotinamide and ADP-ribose of NAD+; and (3) the third part, the acetyl group of the substrate is transferred to the ADP-ribose *via* SIRT2 catalysis, after which two products (the deacetylated substrate and the AADPR) are obtained.

## Emerging Regulator

The benefits of SIRT2 modulation by small molecules have been demonstrated in cancer (Chen et al., 2020) and various metabolic (Yang P. H. et al., 2019) and neurodegenerative disorders (Zhang et al., 2020). Gradually, the potential of the unexplored regulator scaffolds in developing SIRT2 ligands has been rationalized. The regulator scaffolds of SIRT2 include two main modes, namely, the activators and inhibitors (Villalba and Alcaín, 2012). Almost all sirtuin activators are described for SIRT1 (Villalba and Alcaín, 2012), while SIRT2 is rarely involved (Dai et al., 2018). However, the inhibitors of SIRT2 have been widely studied.

The core scaffolds of SIRT2 inhibitors are synthesized by morpholine-catalyzed Knoevenagel condensation/lactonization of hydroxy naphthaldehydes with β-keto esters (Figure 2A).

**FIGURE 3:**
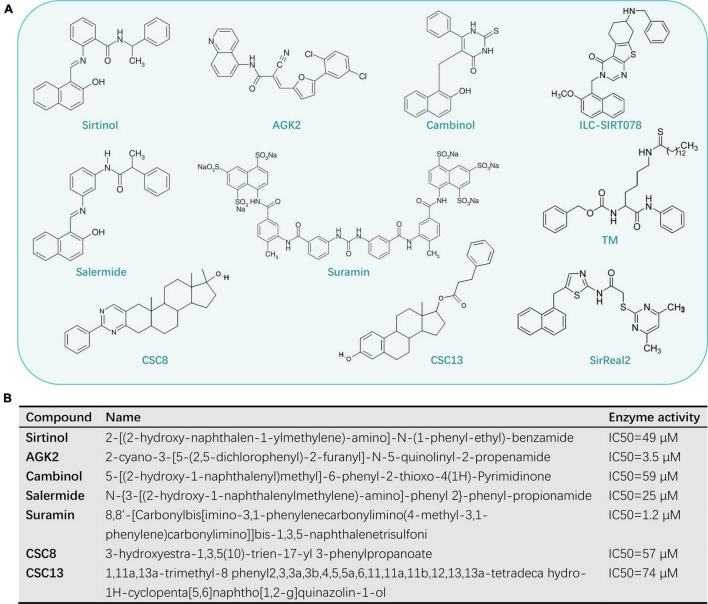
The known SIRT2-selective inhibitors **(A)** and its name of the compound **(B)**.

In view of the variety of SIRT2 functions in cells, they are a druggable class of enzymes that have beneficial effects on various human diseases when selectively activated or inhibited by different molecules. This review focuses on those compounds that act as sirtuin inhibitors, which are potentially useful as therapeutic agents. Several specific inhibitors have been described, including sirtinol (Zhang M. M. et al., 2021), AGK2 (Yu et al., 2018), cambinol (Mahajan et al., 2014), salermide (Mu et al., 2019), suramin (Trapp et al., 2007), CSC8, and CSC13 (Schlicker et al., 2011; Figure 3).

### Sirtinol

Sirtinol is a widely used sirtuin inhibitor that affects the activation of both yeast Sir2p and human SIRT2 *in vitro* (Grozinger et al., 2001; Alcaín and Villalba, 2009). Sirtinol has an IC50 of 38 μM against SIRT2 in an *in vitro* assay, which shows only approximately threefold weaker potency against SIRT1 (Schlicker et al., 2011). Two further analogs, m- and p-sirtinol, have been demonstrated to be more potent than sirtinol against human SIRT2 (Villalba and Alcaín, 2012).

Sirtuin 2 has an important role in deacetylating phosphoenolpyruvate carboxykinase 1 (PEPCK1), a critical enzyme for gluconeogenesis (Jiang et al., 2011; Knyphausen et al., 2016). Also, sirtinol has been previously used to treat type II diabetes (Zhang M. M. et al., 2021). Zhang M. M. et al. (2021) have demonstrated that sirtinol has anti-diabetic effects on cell gluconeogenesis *in vivo* and *in vitro*. In SIRT2-knockdown cells, sirtinol exerts little influence on endogenous PEPCK1 levels (Zhang M. M. et al., 2021). Thus, SIRT2 may be a key target for cetirizine in the treatment of diabetes. In addition, sirtinol administration has also been reported to effectively kill cancer cells when combined with dichloroacetic acid (DCA) and another SIRT2 inhibitor, AGK2 (Ma et al., 2018). AGK2 and sirtinol increase the lysine-acetylation and decrease the serine-phosphorylation of pyruvate dehydrogenase α 1 (PDHA1). Also, the two inhibitors synergize with DCA to further activate PDHA1, which further decreases glucose consumption and lactate production, and increases oxygen consumption rate (OCR) and reactive oxygen species (ROS) generation, which is the function of oxidative phosphorylation (OXPHOS) (Ma et al., 2018). Furthermore, a recent study has demonstrated that lipopolysaccharide (LPS)-stimulated production of tumor necrosis factor-α (TNF-α) and prostaglandin E2 (PGE2) in HAPI rat microglial cells can be inhibited by sirtinol, which protects SH-SY5Y cells from excessive inflammation (Zhang Y. Q. et al., 2021). This research provides theoretical support for further investigation of the therapeutic effect of SIRT2 regulators on LPS-induced neuroinflammation and neurodegeneration.

### AGK2

AGK2, a selective SIRT2 inhibitor, has a calculated IC50 for SIRT2 of 3.5 μM (Alcaín and Villalba, 2009). AGK2 can enter cells easily and act on endogenous SIRT2 in its natural environment. Even at the higher compound concentrations, AGK2 shows only minimal toxicity (Alcaín and Villalba, 2009).

AGK2 reduces the α-synuclein (α-syn) toxicity of dorsomedial dopamine neurons (Outeiro et al., 2007; Suresh and Manjithaya, 2019). Treatment with AGK2 leads to C-6 glioma cell apoptosis *via* the caspase-3-dependent pathway (He et al., 2012; Qin et al., 2014). AGK2 also downregulates the forkhead box O3 (FOXO3a) and mitogen-activated protein kinase (MAPK) signaling pathways, which confer neuroprotection in ischemic stroke (She et al., 2018). In addition, AGK2 treatment decreases the RNAs of hepatitis B virus (HBV), the replication of intermediates, and the secretion of HBeAg and HBsAg, which inhibits HBV replication (Yu et al., 2018). Furthermore, AGK2 reduces the expression of Evans blue pigmentation by inhibiting mast cell activation and lung barrier dysfunction by inhibiting inflammatory responses (Kim Y. Y. et al., 2020).

### Cambinol

Cambinol, a chemically stable compound, shares a β-naphthol pharmacophore with sirtinol (Heltweg et al., 2006; Medda et al., 2009). It is a dual SIRT1/SIRT2 inhibitor, which inhibits the activity of human SIRT2 deacetylase *in vitro* with an IC50 value of 59 μM (Alcaín and Villalba, 2009).

Cambinol has cytotoxic activity against cancer cells *in vitro*. Other studies have shown that cambinol induces apoptosis through the hyperacetylation of BCL6 and p53, which inhibits the growth of Burkitt lymphoma xenografts (Chen, 2011; Chowdhury et al., 2020). Besides, cambinol affects the survival and migration of cellular through modulating the acetylation of p53 and FoxO1 in HepG2 and Huh7 cells. Furthermore, it has been confirmed that SIRT2 activity blockage can be beneficial during hepatocellular carcinoma therapy (Ceballos et al., 2018).

### Salermide

Salermide, a reverse amide compound histone deacetylase inhibitor (HDACI), has a strong inhibitory effect on SIRT2. For example, salermide inhibits 80% of SIRT2 activity in an *in vitro* assay at 25 μM (Trapp et al., 2007). Also, *in vivo* studies suggest that salermide is well-tolerated by mice at concentrations up to 100 μM (Alcaín and Villalba, 2009).

Moreover, studies have found that inhibition of SIRT2 can induce apoptosis in cancer cells, having a multifaceted role in regulating autophagy (Kim et al., 2011). Salermide upregulates heat shock protein family A (Hsp70) member 5 (HSPA5) acetylation and induces pro-survival autophagy *via* the ATF4-DDIT4-mTORC1 axis in human lung cancer cells (Mu et al., 2019). In addition, salermide induces cell death and p53 acetylation by targeting SIRT2 (Peck et al., 2010).

### Suramin

Suramin is a potent inhibitor of SIRT2 NAD+ dependent deacetylase activity, with an IC50 value for SIRT2 of 1.15 μM (Trapp et al., 2007). It is also a polyanionic naphthylurea with antiproliferative and antiviral activity (Alcaín and Villalba, 2009). Nevertheless, the severe neurotoxicity and other systemic side effects of suramin seriously hinder its application in therapeutic treatment (Peltier and Russell, 2002).

### CSC8 and CSC13

CSC8 and CSC13 are SIRT2 inhibitors. The virtual docking of the compound library into the peptide-binding pockets of SIRT2 crystal structures is exploited, which yields compounds discriminating between different isoforms. In activity assays, two compounds show the largest effects in the screen for further characterization. The compounds CSC8 and CSC13 inhibit SIRT2 to ∼80% in the screen (Schlicker et al., 2011). So, CSC8 and CSC13 with micromolar potency and high specificity for SIRT2 are the novel sirtuin inhibitors that were developed based on structure (Botta et al., 2012).

## Inflammatory Factors With Neuroinflammation

Neuroinflammation is considered a critical factor in the progression of multiple neurological diseases (Bai et al., 2021). The inflammatory factors can rapidly activate inflammatory cells and promote the infiltration of inflammatory cells in the injured area, which aggravates brain damage and advances disease progression (Jayaraj et al., 2019).

Sirtuin 2 protects the CNS by reducing excessive inflammation. A previous study found that SIRT2 regulators effectively inhibited LPS (5 ng/mL)-stimulated TNF- and PGE2 production in HAPI rat microglial cells and protected SH-SY5Y human neuroblastoma cells from excessive inflammation (Zhang Y. Q. et al., 2021). In non-neuronal cells, SIRT2 has been shown to function as a tubulin deacetylase and a key regulator of cell division and differentiation. Moreover, the distribution and function of the SIRT2 microtubule (MT) deacetylase are related to the differentiation of cells (Maxwell et al., 2011). Studies have also demonstrated that isoform 3 of SIRT2 is an age-dependent accumulation protein in the CNS (Sola-Sevilla et al., 2021). Also, a preclinical study found a significant increase in the pro-inflammatory cytokine interleukin-1β (IL-1β) in the model mice overexpressing SIRT2.3 (Sola-Sevilla et al., 2021). The most recent study discovered that reduced levels of the intermediate filament protein glial fibrillary acidic protein (GFAP), IL-1β, IL-6, and TNF-α indicated that early SIRT2 inhibition prevents neuroinflammation, which explains the improvement in cognitive deficits shown by 33i-treated senescence accelerated mouse prone 8 (SAMP8) mice (Diaz-Perdigon et al., 2020). Moreover, SIRT2 in microglia can prevent N-methyl-D-aspartic (NMDA)-mediated excitotoxicity in hippocampal slices by resisting the inflammatory signal from LPS (Sade Almeida et al., 2020). In addition, a study found that AGK2 suppresses the expression of inflammatory cytokines like inducible nitric oxide synthase (iNOS), TNF-α, and IL-1β in BV2 mouse microglial cells induced with LPS (Jiao et al., 2020). Also, the same study indicated that AGK2 could reduce the increase of phosphorylation p38, c-Jun N-terminal kinase (JNK), and extracellular signal-regulated kinase (ERK) (Jiao et al., 2020). Besides, AK7, an inhibitor of SIRT2, can inhibit sevoflurane (3%)-induced neuroinflammation and microglial activation by switching microglia from the M1 to M2 phenotype (Wu et al., 2020). The expression of forkhead box P3 (Foxp3), immunosuppression-associated molecules in Treg cells, can be promoted by inhibiting the activity of SIRT2 (Shu et al., 2019). Also, the anti-inflammatory effect of Treg cells on pro-inflammatory macrophages is weakened by SIRT2 using a lentiviral system (Shu et al., 2019). The nuclear factor-kappa B (NF-κB) drives the transcription of pro-inflammatory mediators involved in the terminal effector pathways (Liu X. X. et al., 2019). SIRT2 inhibition increases acetylation and nuclear translocation of NF-κB p65 protein, resulting in the up-regulation of NF-κB targets such as aquaporin 4 (AQP4), matrix metalloproteinases 9 (MMP-9), pro-inflammatory cytokines and chemokines, which exacerbates neuroinflammation (Yuan et al., 2016; Zhang and Chi, 2018). Therefore, the overexpression of SIRT2 alleviates neuroinflammation through the deacetylation of NF-κB.

Sirtuin 2 protects against traumatic brain injury (TBI) by regulating chemokines. Studies have found that secondary injury occurs after TBI, resulting in chronic and progressive neurodegenerative changes (Thapa et al., 2021). These mechanisms are strongly related to microglial-mediated neuroinflammation (Aires et al., 2021). In the patients with severe TBI under neurointensive care, high levels of endothelial chemokine (C-X-C motif) ligand 1 (CXCL1), CXCL10, metaphase chromosome protein 1 (MCP1), MCP2, and IL-8, as well as low levels of the chemokine CC-chemokine ligand 28 (CCL28) and MCP4 have been found (Dyhrfort et al., 2019). Besides, cross-correlation analysis revealed that leukemia inhibitory factor (LIF) and CXCL5 have a central role in the inflammatory cascade (Dyhrfort et al., 2019). Whether SIRT2 can regulate these chemokines and participate in the secondary injury of neuroinflammation after TBI should be addressed by future studies (Dyhrfort et al., 2019).

Sirtuin 2 protects spinal muscular atrophy and Charcot-Marie-Tooth disease by regulating the deacetylation of microrchidia 2 (MORC2), an effector of epigenetic silencing. Missense mutations in MORC2 cause neuropathies, including spinal muscular atrophy and Charcot-Marie-Tooth disease (Douse et al., 2018). In addition, SIRT2 is also the main deacetylase of MORC2 deacetylation (Liu et al., 2020). Knock-down of SIRT2 using siRNAs resulted in an increase in k767AC of MORC2, which further suggested that ectopic expression of SIRT2 reduces the k767AC of MORC2 (Liu et al., 2020). Also, the deacetylase activity of SIRT2 is necessary for the deacetylation of MORC2 (Liu et al., 2020). Therefore, SIRT2 has a potential role in the deacetylation of MORC2 to protect against the excessive neuroinflammation-related diseases.

## Sirtuin 2 and Microglia Physiopathology

Microglial cells, which are the immunocompetent cells and specific to the CNS, have emerged as crucial players in neuroinflammatory conditions (Rodrigues et al., 2018; Ahmad et al., 2022). In addition, studies have shown that the deacetylases SIRT2 can exert its neuroprotective effects through the reduction in neuroinflammation in the CNS, attenuating the microglial response, and releasing pro-inflammatory cytokines (Zhang Y. Q. et al., 2021).

Sirtuin 2 has an important role in the LPS-induced activation of BV2 microglia (Chen et al., 2015; Belarbi et al., 2020). The treatment of BV2 microglia with LPS increases nitric oxide (NO), iNOS, TNF-α, and IL-6 expression, indicating that microglia are activated. SIRT2 silencing significantly reversed this process in microglia cells (Chen et al., 2015). Besides, it was found that SIRT2 mediates the significant increase of the intracellular ATP level and the Akt phosphorylation in BV2 microglia induced by NAD and NADH, while SIRT2 siRNA and the SIRT2 inhibitor AGK2 can reverse this process (Zhang et al., 2018).

Similar research found that the administration of SIRT2 deacetylase inhibitors (AGK2, AK-1, or AK-7) significantly promotes the dectin-induced expression of proinflammatory genes in mouse microglia (Sun et al., 2021). SIRT2 modulators (sirtinol, AGK2) reduce LPS-induced excessive inflammation in HAPI microglial cells and protect SH-SY5Y neuronal cells *in vitro* (Zhang Y. Q. et al., 2021). In addition, the SIRT2 inhibitor AGK2 alleviates LPS-induced neuroinflammation by regulating MAPK phosphatase-1 (MKP-1) in BV2 microglial cells (Jiao et al., 2020). AK7, an inhibitor of SIRT2, attenuates sevoflurane-induced learning and memory deficits in developing rats *via* modulating neuroinflammation and microglial activation (Wu et al., 2020).

It was also observed that SIRT2 protein was mainly expressed in the cytoplasm of neurons but not in astrocytes and microglia (Xie et al., 2017). In this regard, future studies are needed to further explore the role of SIRT2 in microglia physiopathology.

## Sirtuin 2 and Neuroinflammatory Disorders

**FIGURE 4:**
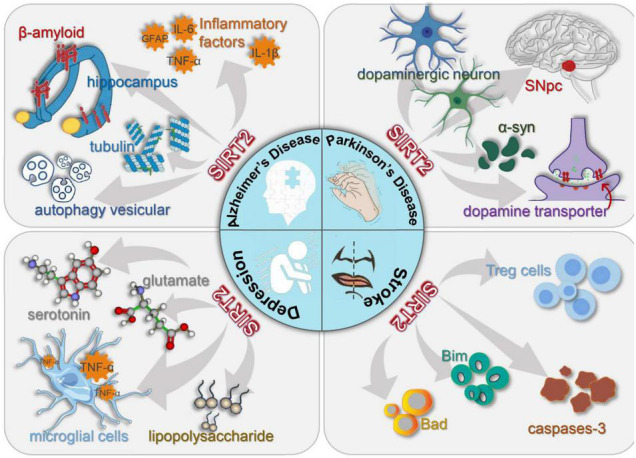
Schematic diagram of the interactions of distinct cell types in four diseases.

The nervous system is known as one of the most complex systems in the human body (Aslani et al., 2021). Lesions of the nervous system seriously affect the quality of life in humans (Fang et al., 2020). With the development of neurobiology, the role of neuroinflammation in nervous system disorders has been widely investigated. Studies have shown that neuroinflammation has a critical role in the pathogenesis of neurological disorders (Jayaraj et al., 2019; Mukhara et al., 2020; de Brito Toscano et al., 2021). SIRT2, a type of NAD+ dependent deacetylases, is predominantly expressed in the cytoplasm of cells in the mammalian CNS (Ma et al., 2020; Chen et al., 2021), which suggests that SIRT2 has a significant role in diseases with neuroinflammatory components (Wang et al., 2016, 2020; Figure 4 and Table 1).

**TABLE 1:** The targets and function of SIRT2 involved in neuroinflammatory disorders.

	Model	Inhibitor	Dose	Target	Functions	References
Alzheimer’s disease	SAMP8 mouse	33i	5 mg/kg, once/day, intraperitoneally (i.p.)	IL-1β, IL-6, GFAP, TNF-α	Prevent neuroinflammation at early stage	Diaz-Perdigon et al., 2020
	APP/PS1 mouse	AK-7	100 mg/kg, twice/day, intraperitoneally (i.p.)	–	Decrease the production of β-amyloid (Aβ) Induce tubulin acetylation Strengthen autophagic trafficking	Wang et al., 2020
Parkinson’s disease	C57BL/6 mouse	miR-212-5p	–	LC3B, p62	Prevent the loss of dopaminergic neuron Prevent the reduction of DAT	Esteves et al., 2018
Depression	C57BL/6 mouse	33i	5–15 mg/kg	Serotonin, GluA1, GluN2B, GluN2A	Increase the serotonin levels and glutamate receptor subunits	Erburu et al., 2017
	HAPI rat microglial cells	Sirtinol, AGK2	20, 10 μM, 0.1 μM	TNF-α, PGE2	Restrain the levels of TNF-α and PGE2 Control inflammation in SH-SY5Y cells	Zhang Y. Q. et al., 2021
Stroke	MCAO mouse Primary neuron-enriched cell	AK1, AGK2	30 μM, 30 μM/1 mg/kg	Caspase-3, Bim, Bad	Attenuate the death of apoptotic cell caused by oxygen-glucose deprivation	She et al., 2018
	GFP+ Treg cells	AGK2	10 μM	HIF-1α	Weaken the anti-inflammatory effect of Treg cells	Wu et al., 2020

### Alzheimer’s Disease

Alzheimer’s disease is the most common cause of dementia globally (Kim S. et al., 2020). Its incidence has been increasing over the years, especially due to the aging of the population, bringing a heavy burden to individuals and society (Soria Lopez et al., 2019). Typical characteristics of AD progress are two hallmark pathologies: β-amyloid (Aβ) plaque deposition and neurofibrillary tangles of hyperphosphorylated tau (Weller and Budson, 2018). Besides the two pathologies, sustained neuroinflammation is also included in the process of AD, which involves the activation of microglia and astrocytes (Laurent et al., 2018).

A previous study found that microglial SIRT2 has a protective role in amnesic deficits associated with neuroinflammation (Sade Almeida et al., 2020). *In vivo* study, LPS significantly reduced the long-term potentiation (LTP) of hippocampal slices in microglia-specific SIRT2 deficient mice (Sade Almeida et al., 2020), while NMDA receptor antagonists restored the LTP values. The results suggest that microglial SIRT2 can prevent NMDA-mediated excitotoxicity in hippocampal slices in response to an inflammatory signal (Sade Almeida et al., 2020).

Another study found that early inhibition of SIRT2 prevents excessive neuroinflammation by decreasing the levels of IL-1β, IL-6, GFAP, and TNF-α, which suggests that SIRT2 is an emerging target for improving excessive neuroinflammation or AD progression (Diaz-Perdigon et al., 2020). A recent study showed that the pharmacological inactivation of SIRT2 exhibits a protective role in AD. For example, in the amyloid precursor protein/presenilin 1 transgenic mouse, the inhibitor of SIRT2 improved cognitive impairment and decreased the production of Aβ in the hippocampus (Wang et al., 2020). A similar protective role of SIRT2 for AD was also shown in another study (Zhang et al., 2020). AK1, an inhibitor of SIRT2, increases the acetylation level of tubulin, the completion of autophagy vesicular, and the aggregation of Aβ in AD cell models. It has been clarified that the inhibition of SIRT2 plays a beneficial role in inducing tubulin acetylation and strengthening autophagic trafficking (Zhang et al., 2020). Other evidence also suggests SIRT2 as a novel target for controlling AD (Jêśko et al., 2017; Silva et al., 2017; Shen et al., 2020).

### Parkinson’s Disease

Parkinson’s disease, a progressive neurodegenerative disease characterized by tremors and bradykinesia, causes heavy losses in productivity and medical resources (Hayes, 2019). The loss of dopaminergic neurons in the substantia nigra striatal pathway and the formation of neuronal inclusions are the main causes of PD (Xie et al., 2021). Yet, recent data have suggested chronic neuroinflammation as another feature of PD (Belarbi et al., 2020).

Some evidence suggests that SIRT2 is an emerging target in controlling PD by participating in the processes of PD pathogenesis, including the aggregation of α-syn, inflammation, oxidative stress, autophagy, and microtubule function (Singh et al., 2021). For example, a recent study found that SIRT2 inhibition exhibited the effects of neuroprotection and anti-inflammation *in vitro* (Liu Y. M. et al., 2019). Meanwhile, the expression level of SIRT2 in degenerating SNpc in the brain of PD was also mentioned (Liu Y. M. et al., 2019), suggesting that SIRT2 might be a potential target in treating PD.

To specifically address the role of SIRT2 in sporadic PD, a SIRT2 knock-out mouse was used to verify the pathogenic role of SIRT2. The SIRT2 knock-out mouse, which is cultured by the neurotoxin 1-methyl-4-phenylpyridinium (MPP+), induced a decrease in mitochondrial membrane potential in mesencephalic neuronal cells (Esteves et al., 2018). The knock-out of SIRT2 deacetylase enhanced α-tubulin acetylation and facilitated the trafficking and clearance of misfolded proteins (Esteves et al., 2018). These results indicated that mitochondrial, microtubule, and autophagy dysfunction are involved in neurodegeneration observed in sporadic PD.

Furthermore, studies have shown that inhibiting SIRT2 activity *via* miR-212-5p transfection can prevent dopaminergic neuron loss and reduce dopamine transporter (DAT), implying that miR-212-5p can control PD by targeting SIRT2 (Sun et al., 2018). These studies all indicate that SIRT2 has an important role in PD.

### Depression

Depression is a common mental disorder characterized by low mood, poor cognition, and anhedonia as its core symptoms (Zheng et al., 2019). Currently, depression is one of the essential contributors to disability globally and one of the leading causes of the global burden of disease (Yao et al., 2019; Sarno et al., 2021). It has been estimated that depression will become the primary factor of disability by 2030 (Subermaniam et al., 2020).

Depression, as a neuropsychiatric disease, also belongs to the category of neuroinflammatory disorders. Among the mainstream hypotheses of the cause of depression, the hypothesis of immune and inflammation has been a hot spot in studying its pathological mechanism (Sharma, 2016; Leonard, 2018; Beurel et al., 2020). Polymorphism studies have shown that SIRT2-related genes are associated with postpartum depression (Luo et al., 2020) and AD depression (Porcelli et al., 2013).

A previous study found that 33i, a selective SIRT2 inhibitor, led to the increase of serotonin levels and glutamate receptor subunits, inducing an antidepressant-like action in the chronic mild stress (CMS) model of depression (Erburu et al., 2017). Another study found that the SIRT2 inhibitors sirtinol and AGK2 significantly decrease the secretion of TNF-α and reduce the production of PGE2 in LPS-stimulated HAPI rat microglial cells (Zhang Y. Q. et al., 2021). In addition, the pretreatment of Sirtinol and AGK2 protects the cells from LPS-stimulated HAPI supernatant in differentiated SH-SY5Y cells (Zhang Y. Q. et al., 2021). SIRT2 inhibition can induce an antidepressant-like action. Moreover, as new antidepressant agents, 33i, sirtinol, and AGK2 have the therapeutic potential to treat depression.

### Stroke

Stroke with a high lifelong disability and fatality rate is one of the most common diseases in modern society (Campbell and Khatri, 2020). It is an acute cerebrovascular disease, resulting in brain tissue damage due to the sudden rupture of blood vessels in the brain (hemorrhagic strokes) (Saand et al., 2019) or vascular occlusion preventing blood from flowing into the brain (ischemic strokes) (Patel et al., 2020). Ischemic stroke is the most common type of stroke (Wei et al., 2020).

Recent studies suggest that secondary neuroinflammation promotes further brain damage, leading to cell death in patients with acute stroke (Jayaraj et al., 2019; Huang et al., 2020). Conversely, the secretion of inflammatory factors is also beneficial as it promotes immune recovery (Jayaraj et al., 2019). SIRT2 has a vital role in stroke as well. It was found that the downregulation of SIRT2 protects the mouse brain against ischemic stroke (Xie et al., 2017). In addition, SIRT2 serves as a risk and prognosis marker for acute ischemic stroke in clinical practice (Zhang et al., 2021b).

Sirtuin 2 induces neuronal cell death in ischemic stroke. Previous studies reported that the SIRT2 inhibitors AK1 and AGK2 could reduce the cleaved caspase-3, Bim, and Bad, by attenuating apoptotic cell death caused by oxygen-glucose deprivation (She et al., 2018). Moreover, the neuroprotective effect of SIRT2 inhibition on ischemic stroke is modulated *via* the downregulation of the protein kinase B (AKT)/FOXO3a axis and the MAPK pathways (She et al., 2018). Another study showed that the anti-inflammatory effect of Treg cells was weakened by SIRT2 on pro-inflammatory macrophages, which indirectly impacted the process of stroke in the model of middle cerebral artery occlusion (Shu et al., 2019).

## Conclusion

In this review, the latest reports on the role of SIRT2 in regulating neuroinflammatory disorders were summarized and highlighted. As one of the most special sirtuins in the CNS, SIRT2 regulates multiple biological functions by NAD+ dependent deacetylation effects, including senescence, myelin formation, autophagy, and inflammation. We focused on the immunomodulatory effects of SIRT2 in the immunology response through molecules of immune regulation of neuroinflammatory disorders.

The immunomodulatory properties and the therapeutic potential of SIRT2 have been demonstrated *in vivo* or *in vitro* models of neuroinflammatory disorders. Moreover, previous studies have indicated that SIRT2 modulates immune balance in nervous system diseases *via* key molecules in different cellular signaling pathways. Herein, we summarized terminal effector molecules of the inflammatory response and reviewed the specific inhibitors of SIRT2, while the specific mechanisms of SIRT2 were inversely verified by relevant molecular biology experiments. Also, the regulatory details of different diseases with neuroinflammatory components, including neurodegenerative and neuropsychiatric diseases, were summed up to a certain extent.

Although the regulatory mechanism of SIRT2 in neuroinflammatory disorders has been studied, some questions still remain. First, the molecular regulating mechanism of SIRT2 has not yet been elaborated. Second, due to the lack of effective activators, it is difficult to assess the potency of SIRT2 inhibitors. Moreover, clinical evidence of immunomodulatory and immune-balancing effects of SIRT2 is lacking. Therefore, further studies should focus on investigating precise targets of SIRT2, which will contribute to developing treatment strategies for neuroinflammatory disorders.

With the state-of-art methods for studying SIRT2 that are expected to be available in the future, studies with an interdisciplinary perspective will ultimately provide a more comprehensive understanding of SIRT2 during the development of neuroinflammatory disorders. We hope this review will draw more attention to preventing and treating neuroinflammatory disorders by targeting SIRT2.

## Author Contributions

ZF conceived and designed the framework of the review, performed literature research and sorting, analyzed most of the data, prepared the figures, and wrote the manuscript. LB conceived and designed the framework of the review, supervised the execution of this project, and contributed to data interpretation and manuscript preparation. Both authors read and approved the final manuscript.

## Conflict of Interest

The authors declare that the research was conducted in the absence of any commercial or financial relationships that could be construed as a potential conflict of interest.

## Publisher’s Note

All claims expressed in this article are solely those of the authors and do not necessarily represent those of their affiliated organizations, or those of the publisher, the editors and the reviewers. Any product that may be evaluated in this article, or claim that may be made by its manufacturer, is not guaranteed or endorsed by the publisher.

## Abbreviations

CNS: central nervous system
SIRT2: sirtuin 2
NAD: nicotinamide adenine dinucleotide
HDACs: histone deacetylases
AADPR: the 2 ′ -O-acetyl-ADP-ribose
PEPCK1: phosphoenolpyruvate carboxykinase 1
DCA: dichloroacetic acid
PDHA1: pyruvate dehydrogenase α 1
OCR: oxygen consumption rate
ROS: reactive oxygen species
OXPHOS: oxidative phosphorylation
LPS: lipopolysaccharide
TNF- α: tumor necrosis factor- α
PGE2: prostaglandin E2
α -syn: α -synuclein
FOXO3a: forkhead box O3
MAPK: mitogen-activated protein kinase
HBV: hepatitis B virus
HSPA5: heat shock protein family A (Hsp70) member 5
IL-1 β: interleukin-1 β
GFAP: glial fibrillary acidic protein
SAMP8: senescence accelerated mouse prone 8
NMDA: N-methyl -D-aspartic
iNOS: inducible nitric oxide synthase
JNK: c-JunN-terminal kinase
ERK: extracellular signal-regulated kinase
Foxp3: forkhead box P3
NF- κ B: nuclear factor-kappa B
AQP4: aquaporin 4
MMP-9: matrix metalloproteinases 9
TBI: traumatic brain injury
CXCL1: chemokine (C-X-C motif) ligand 1
MCP1: metaphase chromosome protein 1
CCL28: CC-chemokine ligand 28
LIF: leukemia inhibitory factor
MORC2: microrchidia 2
NO: nitric oxide
MKP-1: MAPK phosphatase-1
AD: Alzheimer’s disease
A β: β -amyloid
LTP: long-term potentiation
PD: Parkinson’s disease
DAT: dopamine transporter
MPP+: neurotoxin 1-methyl-4-phenylpyridinium
CMS: chronic mild stress
AKT: protein kinase B.

## References

[B1] Ahmad M. A. Kareem O. Khushtar M. Akbar M. Haque M. R. Iqubal A. (2022). Neuroinflammation: A Potential Risk for Dementia. *Int. J. Mol. Sci.* 23:616. 10.3390/ijms23020616 35054805 PMC8775769

[B2] Aires I. D. Ribeiro-Rodrigues T. Boia R. Ferreira-Rodrigues M. Girão H. Ambrósio A. F. (2021). Microglial Extracellular Vesicles as Vehicles for Neurodegeneration Spreading. *Biomolecules* 11:770. 10.3390/biom11060770 34063832 PMC8224033

[B3] Akbari Z. Reisi P. Torkaman-Boutorabi A. Farahmandfar M. (2020). Effect of Pentoxifylline on Apoptotic-Related Gene Expression Profile, Learning and Memory Impairment Induced by Systemic Lipopolysaccharide Administration in the Rat Hippocampus. *Int. J. Prev. Med.* 11 151. 10.4103/ijpvm.IJPVM_170_19 33209221 PMC7643573

[B4] Alcaín F. J. Villalba J. M. (2009). Sirtuin inhibitors. *Expert Opin. Ther. Pat.* 19 283–294. 10.1517/13543770902755111 19441904

[B5] Armstrong R. A. (2019). Risk factors for Alzheimer’s disease. *Folia Neuropathol.* 57 87–105. 10.5114/fn.2019.85929 31556570

[B6] Aslani M. Mortazavi-Jahromi S. S. Mirshafiey A. (2021). Efficient roles of miR-146a in cellular and molecular mechanisms of neuroinflammatory disorders: An effectual review in neuroimmunology. *Immunol. Lett.* 238 1–20. 10.1016/j.imlet.2021.07.004 34293378

[B7] Bai Y. M. Feng Y. L. Jiang B. Yang Y. Pei Z. W. Yang Q. (2021). The Role of Exercise in Reducing Hyperlipidemia-Induced Neuronal Damage in Apolipoprotein E-Deficient Mice. *Biomed. Res. Int.* 2021:5512518. 10.1155/2021/5512518 34409103 PMC8367587

[B8] Belarbi K. Cuvelier E. Bonte M. A. Desplanque M. Gressier B. Devos D. (2020). Glycosphingolipids and neuroinflammation in Parkinson’s disease. *Mol. Neurodegener.* 15:59. 10.1186/s13024-020-00408-1 33069254 PMC7568394

[B9] Beurel E. Toups M. Nemeroff C. B. (2020). The Bidirectional Relationship of Depression and Inflammation: Double Trouble. *Neuron* 107 234–256. 10.1016/j.neuron.2020.06.002 32553197 PMC7381373

[B10] Botta G. De Santis L. P. Saladino R. (2012). Current advances in the synthesis and antitumoral activity of SIRT1-2 inhibitors by modulation of p53 and pro-apoptotic proteins. *Curr. Med. Chem.* 19 5871–5884. 10.2174/092986712804143303 22998567

[B11] Campbell B. C. V. Khatri P. (2020). Stroke. *Lancet* 396 129–142. 10.1016/s0140-6736(20)31179-x 32653056

[B12] Carafa V. Altucci L. Nebbioso A. (2019). Dual Tumor Suppressor and Tumor Promoter Action of Sirtuins in Determining Malignant Phenotype. *Front. Pharmacol.* 10:38. 10.3389/fphar.2019.00038 30761005 PMC6363704

[B13] Carafa V. Nebbioso A. Altucci L. (2012). Sirtuins and disease: the road ahead. *Front. Pharmacol.* 3:4. 10.3389/fphar.2012.00004 22319497 PMC3269041

[B14] Ceballos M. P. Decándido G. Quiroga A. D. Comanzo C. G. Livore V. I. Lorenzetti F. (2018). Inhibition of sirtuins 1 and 2 impairs cell survival and migration and modulates the expression of P-glycoprotein and MRP3 in hepatocellular carcinoma cell lines. *Toxicol. Lett.* 289 63–74. 10.1016/j.toxlet.2018.03.011 29545174

[B15] Chen G. Y. Huang P. Hu C. (2020). The role of SIRT2 in cancer: A novel therapeutic target. *Int. J. Cancer* 147 3297–3304. 10.1002/ijc.33118 32449165

[B16] Chen H. Y. Wu D. H. Ding X. T. Ying W. H. (2015). SIRT2 is required for lipopolysaccharide-induced activation of BV2 microglia. *Neuroreport* 26 88–93. 10.1097/WNR.0000000000000305 25536118

[B17] Chen L. (2011). Medicinal chemistry of sirtuin inhibitors. *Curr. Med. Chem.* 18 1936–1946. 10.2174/09298671179559005 21517778

[B18] Chen X. Q. Lu W. M. Wu D. H. (2021). Sirtuin 2 (SIRT2): Confusing Roles in the Pathophysiology of Neurological Disorders. *Front. Neurosci.* 15:614107. 10.3389/fnins.2021.614107 34108853 PMC8180884

[B19] Chowdhury S. Sripathy S. Webster A. Park A. Lao U. Hsu J. H. (2020). Discovery of Selective SIRT2 Inhibitors as Therapeutic Agents in B-Cell Lymphoma and Other Malignancies. *Molecules* 25:455. 10.3390/molecules25030455 31973227 PMC7036909

[B20] Dai H. Sinclair D. A. Ellis J. L. Steegborn C. (2018). Sirtuin activators and inhibitors: Promises, achievements, and challenges. *Pharmacol. Ther.* 188 140–154. 10.1016/j.pharmthera.2018.03.004 29577959 PMC6342514

[B21] de Brito Toscano E. C. Rocha N. P. Lopes B. N. A. Suemoto C. K. Teixeira A. L. (2021). Neuroinflammation in Alzheimer’s disease: focus on NLRP1 and NLRP3 inflammasomes. *Curr. Protein Pept. Sci.* 22 584–598. 10.2174/1389203722666210916141436 34530705

[B22] Diaz-Perdigon T. Belloch F. B. Ricobaraza A. Elboray E. E. Suzuki T. Tordera R. M. (2020). Early sirtuin 2 inhibition prevents age-related cognitive decline in a senescence-accelerated mouse model. *Neuropsychopharmacology* 45 347–357. 10.1038/s41386-019-0503-8 31471557 PMC6901465

[B23] Douse C. H. Bloor S. Liu Y. C. Shamin M. Tchasovnikarova I. A. Timms R. T. (2018). Neuropathic MORC2 mutations perturb GHKL ATPase dimerization dynamics and epigenetic silencing by multiple structural mechanisms. *Nat. Commun.* 9:651. 10.1038/s41467-018-03045-x 29440755 PMC5811534

[B24] Dyhrfort P. Shen Q. J. Clausen F. Thulin M. Enblad P. Kamali-Moghaddam M. (2019). Monitoring of Protein Biomarkers of Inflammation in Human Traumatic Brain Injury Using Microdialysis and Proximity Extension Assay Technology in Neurointensive Care. *J. Neurotrauma* 36 2872–2885. 10.1089/neu.2018.6320 31017044 PMC6761596

[B25] Erburu M. Muñoz-Cobo I. Diaz-Perdigon T. Mellini P. Suzuki T. Puerta E. (2017). SIRT2 inhibition modulate glutamate and serotonin systems in the prefrontal cortex and induces antidepressant-like action. *Neuropharmacology* 117 195–208. 10.1016/j.neuropharm.2017.01.033 28185898

[B26] Esteves A. R. Arduíno D. M. Silva D. F. Viana S. D. Pereira F. C. Cardoso S. M. (2018). Mitochondrial Metabolism Regulates Microtubule Acetylome and Autophagy Trough Sirtuin-2: Impact for Parkinson’s Disease. *Mol. Neurobiol.* 55 1440–1462. 10.1007/s12035-017-0420-y 28168426

[B27] Fang X. Zhou X. T. Miao Y. Q. Han Y. W. Wei J. Chen T. T. (2020). Therapeutic effect of GLP-1 engineered strain on mice model of Alzheimer’s disease and Parkinson’s disease. *AMB Express* 10:80. 10.1186/s13568-020-01014-6 32333225 PMC7182653

[B28] Finnin M. S. Donigian J. R. Pavletich N. P. (2001). Structure of the histone deacetylase SIRT2. *Nat. Struct. Biol.* 8 621–625. 10.1038/89668 11427894

[B29] Grabowska W. Sikora E. Bielak-Zmijewska A. Sirtuins. (2017). a promising target in slowing down the ageing process. *Biogerontology* 18 447–476. 10.1007/s10522-017-9685-9 28258519 PMC5514220

[B30] Griciuc A. Tanzi R. E. (2021). The role of innate immune genes in Alzheimer’s disease. *Curr. Opin. Neurol.* 34 228–236. 10.1097/wco.0000000000000911 33560670 PMC7954128

[B31] Grozinger C. M. Chao, Blackwell H. E. Moazed D. Schreiber S. L. (2001). Identification of a class of small molecule inhibitors of the sirtuin family of NAD-dependent deacetylases by phenotypic screening. *J Biol. Chem.* 276 38837–38843. 10.1074/jbc.M106779200 11483616

[B32] Gu J. Y. Chen C. Wang J. Chen T. T. Yao W. J. Yan T. D. (2020). Withaferin A Exerts Preventive Effect on Liver Fibrosis through Oxidative Stress Inhibition in a Sirtuin 3-Dependent Manner. *Oxid Med Cell Longev.* 2020:2452848. 10.1155/2020/2452848 33029279 PMC7532400

[B33] Hayes M. T. (2019). Parkinson’s Disease and Parkinsonism. *Am. J. Med.* 132 802–807. 10.1016/j.amjmed.2019.03.001 30890425

[B34] Hayley S. Hakim A. M. Albert P. R. (2021). Depression, dementia and immune dysregulation. *Brain* 144 746–760. 10.1093/brain/awaa405 33279966 PMC8041341

[B35] He X. Nie H. Hong Y. Y. Sheng C. B. Xia W. L. Ying W. H. (2012). SIRT2 activity is required for the survival of C6 glioma cells. *Biochem. Biophys. Res. Commun.* 417 468–472. 10.1016/j.bbrc.2011.11.141 22166219

[B36] Heltweg B. Gatbonton T. Schuler A. D. Posakony J. Li H. Goehle S. (2006). Antitumor activity of a small-molecule inhibitor of human silent information regulator 2 enzymes. *Cancer Res.* 66 4368–4377. 10.1158/0008-5472.Can-05-3617 16618762

[B37] Hong Y. A. Kim J. E. Jo M. Ko G. J. (2020). The Role of Sirtuins in Kidney Diseases. *Int. J. Mol. Sci.* 21:6686. 10.3390/ijms21186686 32932720 PMC7555196

[B38] Hou B. B. Liu X. M. Zheng F. L. Xu X. Z. Zhang Z. Y. (2014). Molecular cloning, modeling and differential expression of a gene encoding a silent information regulator-like protein from Sporothrix schenckii. *Int. J. Mol. Med.* 33 1415–1422. 10.3892/ijmm.2014.1719 24682409 PMC4056409

[B39] Hou Y. J. Dan X. L. Babbar M. Wei Y. Hasselbalch S. G. Croteau D. L. (2019). Ageing as a risk factor for neurodegenerative disease. *Nat. Rev. Neurol.* 15 565–581. 10.1038/s41582-019-0244-7 31501588

[B40] Huang J. B. Chen N. C. Chen C. L. Fu M. H. Pan H. Y. Hsu C. Y. (2020). Serum Levels of Soluble Triggering Receptor Expressed on Myeloid Cells-1 Associated with the Severity and Outcome of Acute Ischemic Stroke. *J. Clin. Med.* 10:61. 10.3390/jcm10010061 33375339 PMC7795761

[B41] Iadecola C. Buckwalter M. S. Anrather J. (2020). Immune responses to stroke: mechanisms, modulation, and therapeutic potential. *J. Clin. Invest.* 130 2777–2788. 10.1172/jci135530 32391806 PMC7260029

[B42] Jayaraj R. L. Azimullah S. Beiram R. Jalal F. Y. Rosenberg G. A. (2019). Neuroinflammation: friend and foe for ischemic stroke. *J. Neuroinflamm.* 16:142. 10.1186/s12974-019-1516-2 31291966 PMC6617684

[B43] Jêśko H. Wencel P. Strosznajder R. P. Strosznajder J. B. (2017). Sirtuins and Their Roles in Brain Aging and Neurodegenerative Disorders. *Neurochem. Res.* 42 876–890. 10.1007/s11064-016-2110-y 27882448 PMC5357501

[B44] Jia Y. Zhang D. D. Yin H. Li H. R. Du J. Bao H. K. (2021). Ganoderic Acid A Attenuates LPS-Induced Neuroinflammation in BV2 Microglia by Activating Farnesoid X Receptor. *Neurochem. Res.* 46 1725–1736. 10.1007/s11064-021-03303-3 33821438 PMC8187184

[B45] Jiang W. Q. Wang S. W. Xiao M. T. Lin Y. Zhou L. S. Lei Q. Y. (2011). Acetylation regulates gluconeogenesis by promoting PEPCK1 degradation via recruiting the UBR5 ubiquitin ligase. *Mol. Cell* 43 33–44. 10.1016/j.molcel.2011.04.028 21726808 PMC3962309

[B46] Jiao F. Z. Wang Y. Zhang W. B. Zhang H. Y. Chen Q. Wang L. W. (2020). AGK2 Alleviates Lipopolysaccharide Induced Neuroinflammation through Regulation of Mitogen-Activated Protein Kinase Phosphatase-1. *J. Neuroimmune Pharmacol.* 15 196–208. 10.1007/s11481-019-09890-x 31786712

[B47] Karaman B. Alhalabi Z. Swyter S. Mihigo S. O. Andrae-Marobela K. Jung M. (2018). Identification of Bichalcones as Sirtuin Inhibitors by Virtual Screening and In Vitro Testing. *Molecules* 23:416. 10.3390/molecules23020416 29443909 PMC6017733

[B48] Kim H. S. Vassilopoulos A. Wang R. H. Lahusen T. Xiao Z. Xu X. (2011). SIRT2 maintains genome integrity and suppresses tumorigenesis through regulating APC/C activity. *Cancer Cell* 20 487–499. 10.1016/j.ccr.2011.09.004 22014574 PMC3199577

[B49] Kim S. Nam Y. Kim C. Lee H. Hong S. Kim H. S. (2020). Neuroprotective and Anti-Inflammatory Effects of Low-Moderate Dose Ionizing Radiation in Models of Alzheimer’s Disease. *Int. J. Mol. Sci.* 21:3678. 10.3390/ijms21103678 32456197 PMC7279400

[B50] Kim Y. Y. Hur G. Lee S. W. Lee S. J. Lee S. Kim S. H. (2020). AGK2 ameliorates mast cell-mediated allergic airway inflammation and fibrosis by inhibiting FcεRI/TGF-β signaling pathway. *Pharmacol. Res.* 159:105027. 10.1016/j.phrs.2020.105027 32565308

[B51] Knyphausen P. de Boor S. Kuhlmann N. Scislowski L. Extra A. Baldus L. (2016). Insights into Lysine Deacetylation of Natively Folded Substrate Proteins by Sirtuins. *J. Biol. Chem.* 291 14677–14694. 10.1074/jbc.M116.726307 27226597 PMC4938187

[B52] Kwon H. S. Koh S. H. (2020). Neuroinflammation in neurodegenerative disorders: the roles of microglia and astrocytes. *Transl. Neurodegener.* 9:42. 10.1186/s40035-020-00221-2 33239064 PMC7689983

[B53] Laurent C. Buée L. Blum D. (2018). Tau and neuroinflammation: What impact for Alzheimer’s Disease and Tauopathies? *Biomed. J.* 41 21–33. 10.1016/j.bj.2018.01.003 29673549 PMC6138617

[B54] Leonard B. E. (2018). Inflammation and depression: a causal or coincidental link to the pathophysiology? *Acta Neuropsychiatr.* 30 1–16. 10.1017/neu.2016.69 28112061

[B55] Lewis J. E. Singh N. Holmila R. J. Sumer B. D. Williams N. S. Furdui C. M. (2019). Targeting NAD(+) Metabolism to Enhance Radiation Therapy Responses. *Semin. Radiat. Oncol.* 29 6–15. 10.1016/j.semradonc.2018.10.009 30573185 PMC6310039

[B56] Li J. Y. Flick F. Verheugd P. Carloni P. Lüscher B. Rossetti G. (2015). Insight into the Mechanism of Intramolecular Inhibition of the Catalytic Activity of Sirtuin 2 (SIRT2). *PLoS One* 10:e0139095. 10.1371/journal.pone.0139095 26407304 PMC4583397

[B57] Li X. J. Egervari G. Wang Y. G. Berger S. L. Lu Z. M. (2018). Regulation of chromatin and gene expression by metabolic enzymes and metabolites. *Nat. Rev. Mol. Cell Biol.* 19 563–578. 10.1038/s41580-018-0029-7 29930302 PMC6907087

[B58] Lin J. L. Xiong Z. C. Gu J. H. Sun Z. R. Jiang S. Fan D. W. (2021). Sirtuins: Potential Therapeutic Targets for Defense against Oxidative Stress in Spinal Cord Injury. *Oxid. Med. Cell Longev.* 2021:7207692. 10.1155/2021/7207692 34257819 PMC8249122

[B59] Liu H. Y. Liu Y. Y. Yang F. Zhang L. Zhang F. L. Hu X. (2020). Acetylation of MORC2 by NAT10 regulates cell-cycle checkpoint control and resistance to DNA-damaging chemotherapy and radiotherapy in breast cancer. *Nucleic Acids Res.* 48 3638–3656. 10.1093/nar/gkaa130 32112098 PMC7144926

[B60] Liu X. X. Liu F. H. Ma Y. F. Li H. R. Ju X. H. Xu J. Q. (2019). Effect of Puerarin, Baicalin and Berberine Hydrochloride on the Regulation of IPEC-J2 Cells Infected with Enterotoxigenic *Escherichia coli*. *Evid. Based Complement Alternat. Med.* 2019:7438593. 10.1155/2019/7438593 30891078 PMC6390247

[B61] Liu Y. M. Zhang Y. Y. Zhu K. H. Chi S. Wang C. Xie A. M. (2019). Emerging Role of Sirtuin 2 in Parkinson’s Disease. *Front. Aging Neurosci.* 11:372. 10.3389/fnagi.2019.00372 31998119 PMC6965030

[B62] Lu W. M. Wang Q. Xu C. Yuan H. H. Fan Q. Chen B. Y. (2021). SUMOylation is essential for Sirt2 tumor-suppressor function in neuroblastoma. *Neoplasia* 23 129–139. 10.1016/j.neo.2020.11.013 33316537 PMC7736920

[B63] Luo S. C. Duan K. M. Fang C. Li D. Y. Zheng S. S. Yang S. Q. (2020). Correlations Between SIRT Genetic Polymorphisms and Postpartum Depressive Symptoms in Chinese Parturients Who Had Undergone Cesarean Section. *Neuropsychiatr. Dis. Treat.* 16 3225–3238. 10.2147/ndt.S278248 33380799 PMC7769146

[B64] Ma L. Maruwge W. Strambi A. D’Arcy P. Pellegrini P. Kis L. (2014). SIRT1 and SIRT2 inhibition impairs pediatric soft tissue sarcoma growth. *Cell Death Dis.* 5:e1483. 10.1038/cddis.2014.385 25341037 PMC4237232

[B65] Ma W. J. Zhao X. P. Wang K. Y. Liu J. J. Huang G. (2018). Dichloroacetic acid (DCA) synergizes with the SIRT2 inhibitor Sirtinol and AGK2 to enhance anti-tumor efficacy in non-small cell lung cancer. *Cancer Biol. Ther.* 19 835–846. 10.1080/15384047.2018.1480281 30067423 PMC6154844

[B66] Ma Y. G. Deng M. Zhao X. Q. Liu M. (2020). Alternatively Polarized Macrophages Regulate the Growth and Differentiation of Ependymal Stem Cells through the SIRT2 Pathway. *Exp. Neurobiol.* 29 150–163. 10.5607/en19078 32408405 PMC7237271

[B67] Mahajan S. S. Scian M. Sripathy S. Posakony J. Lao U. Loe T. K. (2014). Development of pyrazolone and isoxazol-5-one cambinol analogues as sirtuin inhibitors. *J. Med. Chem.* 57 3283–3294. 10.1021/jm4018064 24697269 PMC4002067

[B68] Maida C. D. Norrito R. L. Daidone M. Tuttolomondo A. Pinto A. (2020). Neuroinflammatory Mechanisms in Ischemic Stroke: Focus on Cardioembolic Stroke, Background, and Therapeutic Approaches. *Int. J. Mol. Sci.* 21:6454. 10.3390/ijms21186454 32899616 PMC7555650

[B69] Maxwell M. M. Tomkinson E. M. Nobles J. Wizeman J. W. Amore A. M. Quinti L. (2011). The Sirtuin 2 microtubule deacetylase is an abundant neuronal protein that accumulates in the aging CNS. *Hum. Mol. Genet.* 20 3986–3996. 10.1093/hmg/ddr326 21791548 PMC3203628

[B70] Medda F. Russell R. J. Higgins M. McCarthy A. R. Campbell J. Slawin A. M. (2009). Novel cambinol analogs as sirtuin inhibitors: synthesis, biological evaluation, and rationalization of activity. *J. Med. Chem.* 52 2673–2682. 10.1021/jm8014298 19419202 PMC2691587

[B71] Minten E. V. Kapoor-Vazirani P. Li C. Zhang H. Balakrishnan K. Yu D. S. (2021). SIRT2 promotes BRCA1-BARD1 heterodimerization through deacetylation. *Cell Rep.* 34:108921. 10.1016/j.celrep.2021.108921 33789098 PMC8108010

[B72] Mu N. Lei Y. J. Wang Y. Wang Y. Y. Duan Q. H. Ma G. L. (2019). Inhibition of SIRT1/2 upregulates HSPA5 acetylation and induces pro-survival autophagy via ATF4-DDIT4-mTORC1 axis in human lung cancer cells. *Apoptosis* 24 798–811. 10.1007/s10495-019-01559-3 31321634

[B73] Mukhara D. Oh U. Neigh G. N. (2020). Neuroinflammation. *Handb. Clin. Neurol.* 175 235–259. 10.1016/b978-0-444-64123-6.00017-5 33008528

[B74] North B. J. Verdin E. (2004). Sirtuins: Sir2-related NAD-dependent protein deacetylases. *Genom. Biol.* 5:224. 10.1186/gb-2004-5-5-224 15128440 PMC416462

[B75] Outeiro T. F. Kontopoulos E. Altmann S. M. Kufareva I. Strathearn K. E. Amore A. M. (2007). Sirtuin 2 inhibitors rescue alpha-synuclein-mediated toxicity in models of Parkinson’s disease. *Science* 317 516–519. 10.1126/science.1143780 17588900

[B76] Pascoe M. C. Parker A. G. (2019). Physical activity and exercise as a universal depression prevention in young people: A narrative review. *Early Interv. Psychiatr.* 13 733–739. 10.1111/eip.12737 30302925

[B77] Patel P. Yavagal D. Khandelwal P. (2020). Hyperacute Management of Ischemic Strokes: JACC Focus Seminar. *J. Am. Coll. Cardiol.* 75 1844–1856. 10.1016/j.jacc.2020.03.006 32299596

[B78] Peck B. Chen C. Y. Ho K. K. Di Fruscia P. Myatt S. S. Coombes R. C. (2010). SIRT inhibitors induce cell death and p53 acetylation through targeting both SIRT1 and SIRT2. *Mol. Cancer Ther.* 9 844–855. 10.1158/1535-7163.Mct-09-0971 20371709

[B79] Peltier A. C. Russell J. W. (2002). Recent advances in drug-induced neuropathies. *Curr. Opin. Neurol.* 15 633–638. 10.1097/00019052-200210000-00015 12352008

[B80] Porcelli S. Salfi R. Politis A. Atti A. R. Albani D. Chierchia A. (2013). Association between Sirtuin 2 gene rs10410544 polymorphism and depression in Alzheimer’s disease in two independent European samples. *J. Neural Transm.* 120 1709–1715. 10.1007/s00702-013-1045-6 23712749

[B81] Puigoriol-Illamola D. Martínez-Damas M. Griñán-Ferré C. Pallàs M. (2020). Chronic Mild Stress Modified Epigenetic Mechanisms Leading to Accelerated Senescence and Impaired Cognitive Performance in Mice. *Int. J. Mol. Sci.* 21:1154. 10.3390/ijms21031154 32050516 PMC7037343

[B82] Putaala J. (2020). Ischemic Stroke in Young Adults. *Continuum* 26 386–414. 10.1212/con.0000000000000833 32224758

[B83] Qin B. Panickar K. S. Anderson R. A. (2014). Cinnamon polyphenols regulate S100β, sirtuins, and neuroactive proteins in rat C6 glioma cells. *Nutrition* 30 210–217. 10.1016/j.nut.2013.07.001 24239092

[B84] Ren Y. R. Ye Y. L. Feng Y. Xu T. F. Shen Y. Liu J. (2020). SL010110, a lead compound, inhibits gluconeogenesis via SIRT2-p300-mediated PEPCK1 degradation and improves glucose homeostasis in diabetic mice. *Acta Pharmacol. Sin.* 42 1834–1846. 10.1038/s41401-020-00609-w 33574568 PMC8563938

[B85] Rodrigues A. C. Aires I. D. Vindeirinho J. Boia R. Madeira M. H. Gonçalves F. Q. (2018). Elevated Pressure Changes the Purinergic System of Microglial Cells. *Front. Pharmacol.* 9:16. 10.3389/fphar.2018.00016 29416510 PMC5787565

[B86] Saand A. R. Yu F. Chen J. Chou S. H. (2019). Systemic inflammation in hemorrhagic strokes - A novel neurological sign and therapeutic target? *J. Cereb. Blood Flow Metab.* 39 959–988. 10.1177/0271678x19841443 30961425 PMC6547186

[B87] Sade Almeida J. Vargas M. Fonseca-Gomes J. Tanqueiro S. R. Belo R. F. (2020). Microglial Sirtuin 2 Shapes Long-Term Potentiation in Hippocampal Slices. *Front. Neurosci.* 14:614. 10.3389/fnins.2020.00614 32625056 PMC7315392

[B88] Sarno E. Moeser A. J. Robison A. J. (2021). Neuroimmunology of depression. *Adv. Pharmacol.* 91 259–292. 10.1016/bs.apha.2021.03.004 34099111 PMC8877598

[B89] Satoh A. Stein L. Imai S. (2011). The role of mammalian sirtuins in the regulation of metabolism, aging, and longevity. *Handb. Exp. Pharmacol.* 206 125–162. 10.1007/978-3-642-21631-2_7 21879449 PMC3745303

[B90] Schlicker C. Boanca G. Lakshminarasimhan M. Steegborn C. (2011). Structure-based development of novel sirtuin inhibitors. *Aging* 3 852–872. 10.18632/aging.100388 21937767 PMC3227451

[B91] Schonhoff A. M. Williams G. P. Wallen Z. D. Standaert D. G. Harms A. S. (2020). Innate and adaptive immune responses in Parkinson’s disease. *Prog. Brain Res.* 252 169–216. 10.1016/bs.pbr.2019.10.006 32247364 PMC7185735

[B92] Sedlackova L. Korolchuk V. I. (2020). The crosstalk of NAD. ROS and autophagy in cellular health and ageing. *Biogerontology* 21 381–397. 10.1007/s10522-020-09864-0 32124104 PMC7196094

[B93] Sharma A. (2016). Systems Genomics Support for Immune and Inflammation Hypothesis of Depression. *Curr. Neuropharmacol.* 14 749–758. 10.2174/1570159x14666160106155331 26733279 PMC5050401

[B94] She D. T. Wong L. J. Baik S. H. Arumugam T. V. (2018). SIRT2 Inhibition Confers Neuroprotection by Downregulation of FOXO3a and MAPK Signaling Pathways in Ischemic Stroke. *Mol. Neurobiol.* 55 9188–9203. 10.1007/s12035-018-1058-0 29654491

[B95] Shen Y. Y. Chen L. Y. Zhang S. J. Xie L. Q. (2020). Correlation Between SIRT2 3’UTR Gene Polymorphism and the Susceptibility to Alzheimer’s Disease. *J. Mol. Neurosci.* 70 878–886. 10.1007/s12031-020-01513-y 32124252

[B96] Shu L. Xu C. Q. Yan Z. Y. Yan Y. Jiang S. Z. Wang Y. R. (2019). Post-Stroke Microglia Induce Sirtuin2 Expression to Suppress the Anti-inflammatory Function of Infiltrating Regulatory T Cells. *Inflammation* 42 1968–1979. 10.1007/s10753-019-01057-3 31297748

[B97] Silva D. F. Esteves A. R. Oliveira C. R. Cardoso S. M. (2017). Mitochondrial Metabolism Power SIRT2-Dependent Deficient Traffic Causing Alzheimer’s-Disease Related Pathology. *Mol. Neurobiol.* 54 4021–4040. 10.1007/s12035-016-9951-x 27311773

[B98] Singh A. P. Nigam L. Yadav Y. Shekhar S. Subbarao N. Dey S. (2021). Design and in vitro analysis of SIRT2 inhibitor targeting Parkinson’s disease. *Mol. Divers.* 25 2261–2270. 10.1007/s11030-020-10116-z 32591930

[B99] Sola-Sevilla N. Ricobaraza A. Hernandez-Alcoceba R. Aymerich M. S. Tordera R. M. Puerta E. (2021). Understanding the Potential Role of Sirtuin 2 on Aging: Consequences of SIRT2.3 Overexpression in Senescence. *Int. J. Mol. Sci.* 22:3017. 10.3390/ijms22063107 33803627 PMC8003096

[B100] Soria Lopez J. A. González H. M. Léger G. C. (2019). Alzheimer’s disease. *Handb. Clin. Neurol.* 167 231–255. 10.1016/b978-0-12-804766-8.00013-3 31753135

[B101] Subermaniam K. Yow Y. Y. Lim S. H. Koh O. H. Wong K. H. (2020). Malaysian macroalga Padina australis Hauck attenuates high dose corticosterone-mediated oxidative damage in PC12 cells mimicking the effects of depression. *Saud. J. Biol. Sci.* 27 1435–1445. 10.1016/j.sjbs.2020.04.042 32489279 PMC7254034

[B102] Sun S. F. Han X. J. Li X. T. Song Q. Q. Lu M. Jia M. M. (2018). MicroRNA-212-5p Prevents Dopaminergic Neuron Death by Inhibiting SIRT2 in MPTP-Induced Mouse Model of Parkinson’s Disease. *Front. Mol. Neurosci.* 11:381. 10.3389/fnmol.2018.00381 30364275 PMC6193094

[B103] Sun W. W. Ma X. J. Wang H. P. Du Y. Y. Chen J. W. Hu H. J. (2021). MYO1F regulates antifungal immunity by regulating acetylation of microtubules. *Proc. Natl. Acad. Sci. USA* 118:e2100230118. 10.1073/pnas.2100230118 34301894 PMC8325298

[B104] Suresh S. N. Manjithaya R. (2019). A small molecule autophagy inducer exerts cytoprotection against α-synuclein toxicity. *Eur. J. Pharmacol.* 862:172635. 10.1016/j.ejphar.2019.172635 31491404

[B105] Tatone C. Di Emidio G. Vitti M. Di Carlo M. Santini S. Jr. D’Alessandro A. M. (2015). Sirtuin Functions in Female Fertility: Possible Role in Oxidative Stress and Aging. *Oxid Med Cell Longev* 2015 659687. 10.1155/2015/659687 26075037 PMC4436464

[B106] Thapa K. Khan H. Singh T. G. Kaur A. (2021). Traumatic Brain Injury: Mechanistic Insight on Pathophysiology and Potential Therapeutic Targets. *J. Mol. Neurosci.* 71 1725–1742. 10.1007/s12031-021-01841-7 33956297

[B107] Trapp J. Meier R. Hongwiset D. Kassack M. U. Sippl W. Jung M. (2007). Structure-activity studies on suramin analogues as inhibitors of NAD+-dependent histone deacetylases (sirtuins). *Chem. Med. Chem.* 2 1419–1431. 10.1002/cmdc.200700003 17628866

[B108] Villalba J. M. Alcaín F. J. (2012). Sirtuin activators and inhibitors. *Biofactors* 38 349–359. 10.1002/biof.1032 22730114 PMC3467333

[B109] Wang B. Zhang Y. J. Cao W. Wei X. B. Chen J. Ying W. H. (2016). SIRT2 Plays Significant Roles in Lipopolysaccharides-Induced Neuroinflammation and Brain Injury in Mice. *Neurochem. Res.* 41 2490–2500. 10.1007/s11064-016-1981-2 27350577

[B110] Wang Y. Yang J. Q. Hong T. T. Chen X. J. Cui L. L. (2019). SIRT2: Controversy and multiple roles in disease and physiology. *Ageing Res Rev* 55 100961. 10.1016/j.arr.2019.100961 31505260

[B111] Wang Y. Yang J. Q. Hong T. T. Sun Y. H. Huang H. L. Chen F. (2020). RTN4B-mediated suppression of Sirtuin 2 activity ameliorates β-amyloid pathology and cognitive impairment in Alzheimer’s disease mouse model. *Aging Cell* 19:e13194. 10.1111/acel.13194 32700357 PMC7431833

[B112] Wei Z. Q. Qi X. D. Chen Y. Xia X. S. Zheng B. Y. Sun X. G. (2020). Bioinformatics method combined with logistic regression analysis reveal potentially important miRNAs in ischemic stroke. *Biosci. Rep.* 40:BSR20201154. 10.1042/bsr20201154 32744319 PMC7432999

[B113] Weller J. Budson A. (2018). Current understanding of Alzheimer’s disease diagnosis and treatment. *F1000Res* 7:F1000FacultyRev–1161. 10.12688/f1000research.14506.1 30135715 PMC6073093

[B114] Wu Z. Y. Zhang Y. Zhang Y. N. Zhao P. (2020). Sirtuin 2 Inhibition Attenuates Sevoflurane-Induced Learning and Memory Deficits in Developing Rats via Modulating Microglial Activation. *Cell Mol. Neurobiol.* 40 437–446. 10.1007/s10571-019-00746-9 31713761 PMC11449016

[B115] Xie J. Chen S. T. Bopassa J. C. Banerjee S. (2021). Drosophila tubulin polymerization promoting protein mutants reveal pathological correlates relevant to human Parkinson’s disease. *Sci. Rep.* 11:13614. 10.1038/s41598-021-92738-3 34193896 PMC8245532

[B116] Xie X. Q. Zhang P. Tian B. Chen X. Q. (2017). Downregulation of NAD-Dependent Deacetylase SIRT2 Protects Mouse Brain Against Ischemic Stroke. *Mol. Neurobiol.* 54 7251–7261. 10.1007/s12035-016-0173-z 27796760

[B117] Yang B. M. Figueroa D. M. Hou Y. J. Babbar M. Baringer S. L. Croteau D. L. (2019). NEIL1 stimulates neurogenesis and suppresses neuroinflammation after stress. *Free Radic. Biol. Med.* 141 47–58. 10.1016/j.freeradbiomed.2019.05.037 31175982 PMC7526462

[B118] Yang P. H. Xu C. Reece E. A. Chen X. Zhong J. X. Zhan M. (2019). Tip60- and sirtuin 2-regulated MARCKS acetylation and phosphorylation are required for diabetic embryopathy. *Nat. Commun.* 10:282. 10.1038/s41467-018-08268-6 30655546 PMC6336777

[B119] Yang Q. J. Zhou Y. X. Sun Y. T. Luo Y. Shen Y. Shao A. W. (2020). Will Sirtuins Be Promising Therapeutic Targets for TBI and Associated Neurodegenerative Diseases? *Front. Neurosci.* 14:791. 10.3389/fnins.2020.00791 32848564 PMC7411228

[B120] Yang W. T. Gao F. Zhang P. Pang S. C. Cui Y. H. Liu L. X. (2017). Functional genetic variants within the SIRT2 gene promoter in acute myocardial infarction. *PLoS One* 12:e0176245. 10.1371/journal.pone.0176245 28445509 PMC5406008

[B121] Yang Y. Ding J. Gao Z. G. Wang Z. J. (2017). A variant in SIRT2 gene 3’-UTR is associated with susceptibility to colorectal cancer. *Oncotarget* 8 41021–41025. 10.18632/oncotarget.17460 28514749 PMC5522236

[B122] Yao Y. C. Ding G. Q. Wang L. L. Jin Y. Lin J. W. Zhai Y. J. (2019). Risk Factors for Depression in Empty Nesters: A Cross-Sectional Study in a Coastal City of Zhejiang Province and China. *Int. J. Environ. Res. Public Health* 16:4106. 10.3390/ijerph16214106 31653106 PMC6862174

[B123] Yu H. B. Jiang H. Cheng S. T. Hu Z. W. Ren J. H. Chen J. (2018). AGK2, A SIRT2 Inhibitor, Inhibits Hepatitis B Virus Replication In Vitro And In Vivo. *Int. J. Med. Sci.* 15 1356–1364. 10.7150/ijms.26125 30275764 PMC6158674

[B124] Yuan F. Xu Z. M. Lu L. Y. Nie H. Ding J. Ying W. H. (2016). SIRT2 inhibition exacerbates neuroinflammation and blood-brain barrier disruption in experimental traumatic brain injury by enhancing NF-κB p65 acetylation and activation. *J. Neurochem.* 136 581–593. 10.1111/jnc.13423 26546505

[B125] Zhang J. Wang C. X. Ying W. H. (2018). SIRT2 and Akt mediate NAD+-induced and NADH-induced increases in the intracellular ATP levels of BV2 microglia under basal conditions. *Neuroreport* 29 65–70. 10.1097/WNR.0000000000000876 29189472

[B126] Zhang X. Ameer F. S. Azhar G. Wei J. Y. (2021). Alternative Splicing Increases Sirtuin Gene Family Diversity and Modulates Their Subcellular Localization and Function. *Int. J. Mol. Sci.* 22:473. 10.3390/ijms22020473 33418837 PMC7824890

[B127] Zhang Y. Anoopkumar-Dukie S. Davey A. K. (2021a). SIRT1 and SIRT2 Modulators: Potential Anti-Inflammatory Treatment for Depression? *Biomolecules* 11:353. 10.3390/biom11030353 33669121 PMC7996578

[B128] Zhang Y. Chi D. C. (2018). Overexpression of SIRT2 Alleviates Neuropathic Pain and neuroinflammation Through Deacetylation of Transcription Factor Nuclear Factor-Kappa B. *Inflammation* 41 569–578. 10.1007/s10753-017-0713-3 29222643

[B129] Zhang Y. Yan Q. F. Zhang Y. (2021b). Overexpression of sirtuin 2 and its association with prognosis in acute ischemic stroke patients. *J. Clin Lab. Anal.* 35:e23707. 10.1002/jcla.23707 33616302 PMC8059742

[B130] Zhang Y. Q. Anoopkumar-Dukie S. Arora D. Davey A. K. (2020). Review of the anti-inflammatory effect of SIRT1 and SIRT2 modulators on neurodegenerative diseases. *Eur J Pharmacol* 867 172847. 10.1016/j.ejphar.2019.172847 31812544

[B131] Zhang Y. Q. Anoopkumar-Dukie S. Mallik S. B. Davey A. K. (2021). SIRT1 and SIRT2 modulators reduce LPS-induced inflammation in HAPI microglial cells and protect SH-SY5Y neuronal cells in vitro. *J. Neural Transm.* 128 631–644. 10.1007/s00702-021-02331-1 33821324

[B132] Zhang M. M. Pan Y. D. Dorfman R. G. Yin Y. Y. Zhou Q. Huang S. (2021) . Sirtinol promotes PEPCK1 degradation and inhibits gluconeogenesis by inhibiting deacetylase SIRT2. *Sci. Rep.* 7:7. 10.1038/s41598-017-00035-9 28127057 PMC5428341

[B133] Zheng X. X. Cheng Y. Chen Y. W. Yue Y. S. Li Y. C. Xia S. Z. (2019). Ferulic Acid Improves Depressive-Like Behavior in Prenatally-Stressed Offspring Rats via Anti-Inflammatory Activity and HPA Axis. *Int. J. Mol. Sci.* 20:493. 10.3390/ijms20030493 30678337 PMC6387299

